# The Effect of Auriculotherapy on an Overweight Pregnant Women's Weight-Gaining Pattern: A Randomized Controlled Clinical Trial

**DOI:** 10.1155/2023/7192142

**Published:** 2023-09-28

**Authors:** Malihe Rezvanimagham, Shahla Faal Siahkal, Elham Ebrahimi

**Affiliations:** ^1^Department of Midwifery, School of Nursing & Midwifery, Tehran University of Medical Sciences, Tehran, Iran; ^2^Department of Midwifery, Marand Branch, Islamic Azad University, Marand, Iran; ^3^Department of Reproductive Health Midwifery, School of Nursing & Midwifery, Tehran University of Medical Sciences, Tehran, Iran; ^4^Nursing and Midwifery Care Research Center, School of Nursing & Midwifery, Tehran University of Medical Sciences, Tehran, Iran

## Abstract

**Background:**

A concomitant increase in pregnancy complications has accompanied the growing global trend of excessive weight gain during pregnancy. This study evaluates the effect of ear acupressure (auriculotherapy) on the weight-gaining pattern of overweight women during pregnancy.

**Materials and Methods:**

This study was a single-blinded randomized clinical trial conducted between January and September 2022. This study took place in health centers of Qom University of Medical Sciences in Iran. One-hundred thirty overweight pregnant women were selected by a purposeful sampling method and then divided into two groups by block randomization method. In the intervention group, two seeds were placed in the left ear on the metabolism and stomach points, while two seeds were placed in the right ear on the mouth and appetite points. Participants in the intervention group must press the seeds six times a day, 20 minutes before a meal for five weeks. For the placebo group, the *Vaccaria* seedless label was placed at the same points as the intervention group. A digital scale with an accuracy of 0.1 kg was used to weigh the pregnant women during each visit. Descriptive statistics, independent *T*-test, chi-square, and repeated measure ANOVA (analysis of variance) test were used to check the research objectives.

**Results:**

There was a statistically significant difference between the auriculotherapy and placebo groups immediately after completing the study (1120.68 ± 425.83 vs. 2704.09 ± 344.96 (g); *P* = 0.018), respectively. Also, there was a substantial difference in the weight gain of women two weeks (793.10 ± 278.38 vs. 1090.32 ± 330.31 (g); *P* < 0.001) and four weeks after the intervention (729.31 ± 241.52 vs. 964.51 ± 348.35 (g); *P* < 0.001) between the auriculotherapy and placebo groups. *Discussion*. The results of the present study indicated the effectiveness of auriculotherapy in controlling the weight gain of overweight pregnant women. This treatment could be used as a safe method, with easy access, and low cost in low-risk pregnancies. Trial Registration. This trial is registered with IRCT20200104046002N1.

## 1. Introduction

Obesity and being overweight are common metabolic disorders that impact individual's physical and mental health [1]. Adult overweight and obesity are defined by the World Health Organization (WHO) as follows: Obesity is defined as having a body mass index (BMI) greater than or equal to 30 kg/m^2^ and BMI greater than or equal to 25 kg/m^2^ indicates overweight [2].

Obesity is the most common healthcare problem in women of childbearing age [3]. In 2014, there were approximately 38.9 million overweight and pregnant women with obesity worldwide, of whom approximately 14.6 million were obese [4]. Obesity may be associated with various pregnancy complications such as gestational diabetes, gestational hypertension, and preeclampsia [5] and affects approximately 50% of women in low-income countries [6]. Also, fetal complications, including congenital malformations (heart and neural tube), stillbirth, macrosomia, miscarriage, and preterm delivery, are abundant in these women [7, 8].

Proper pregnancy weight gain may be necessary for pregnant women to achieve safe outcomes of pregnancy, childbirth, as well as long-term health [9]. Therefore, in 2009, the Institute of Medicine (IOM) published revised guidelines for pregnancy weight gain based on the prepregnancy BMI recommended by the WHO for underweight, normal weight, overweight, and obesity regardless of age, race, and ethnicity [10]. The instructions of the Institute of Medicine recommend general weight gain for overweight pregnant women in the range of 6.8–11.3 kg. The purpose of weight gain recommendations during pregnancy is to optimize the outcomes for the mother and baby [11]. Weight control is a significant public health issue that should be considered to improve people's health and reduce medical costs [12]. Despite regular and planned care of pregnant women in Iran, only half achieve the recommended weight during pregnancy [13].

Auriculotherapy (treatment through the ear) is a type of acupuncture that has become a separate and unique alternative treatment method in the last 60 years and was officially recognized by the WHO in 1990 [14, 15]. Ear acupuncture can reduce the pleasure of eating and the feeling of hunger [16] and increase the feeling of satiety [17]. It is also more effective in weight loss than body acupuncture. Therefore, it can be a useful and safe nondrug treatment [18].

In a study conducted by Bradford et al., acupuncture was performed unilaterally on 5 points of the ear of nonpregnant individuals. The results showed that within 30 minutes after treatment, appetite decreased in the intervention group compared to the placebo group. However, the difference was not statistically significant [17]. In the study by Hsieh et al., the ear points were used to treat the weight loss of individuals. The group treated with *Vaccaria* seed showed rapid weight loss in the short term and it was found that acupressure can be a good option in the treatment of overweight and obesity in young nonpregnant adults [19]. The systematic review and meta-analysis performed by Mendonca et al. showed that auriculotherapy may be effective in reducing weight and/or BMI in individuals with overweight or obesity [20].

So far, several studies have investigated the effect of auriculotherapy on problems during pregnancy such as anxiety [21], pain [22], and nausea and vomiting [23]. However, to the best of our knowledge, no study has been conducted on the effect of auriculotherapy on the weight gain of pregnant mothers. As a result, the researchers decided to design a study to investigate the effect of ear acupressure (auriculotherapy) on the weight gain status of overweight women during pregnancy.

## 2. Methods

This study was a single-blind randomized clinical trial with two intervention and placebo groups which was conducted in the selected health centers of Qom city in Iran between January and September 2022. This study was designed according to CONSORT standards. The protocol of this study was published in the International Journal of Travel Medicine and Global Health (IJTMGH) [24].

### 2.1. Participants

In this study, 130 pregnant women referring to selected health centers in Qom (Iran), who were overweight and were between 20 and 24 weeks of pregnancy, and who fulfill the inclusion criteria were invited to participate.

### 2.2. Inclusion Criteria

The inclusion criteria were as follows: BMI 25 to 29.9 kg/m^2^ before pregnancy or during the first trimester of pregnancy, excessive weight gain from the beginning of the 13th week of pregnancy onwards of more than 300 g per week in the second half of pregnancy [25], age 18 to 40 years, singleton pregnancy, gestational age at least 20 full weeks (due to reduced nausea and increased maternal appetite after the end of the first trimester of pregnancy and excessive need of help for these groups of women) and at most 24 full weeks (for meeting the complete duration of five weeks for each women in study groups), and literacy.

### 2.3. Exclusion Criteria

The exclusion criteria were as follows: Reluctance to continue participating in the study for any reason, failure to complete the intervention period due to preterm labor or the other causes, occurrence of any side effects following the intervention, creating new medical conditions during the study period (complications of pregnancy such as gestational diabetes mellitus, hypothyroidism, bleeding, hypertension, preterm labor, rupture, or water bag leakage), using any other weight control method (under the diet of a nutritionist, etc.), terminating the pregnancy for any reason, history of miscarriage, stillbirth and infertility, medical problems affecting body weight (untreated thyroid disease), history of diabetes mellitus type 1 or 2, drug-dependent hypertension, addiction, nutritional problems, chronic disease, kidney disease, anemia, medication, psychiatric illness, history of eating disorders, and ear abnormalities or ear infections.

### 2.4. Ethical Consideration

In this study, the ethical code was obtained from the Nursing and Midwifery Faculty of Tehran University of Medical Sciences (TUMS) Ethics Committee with the Ref. ID: IR.TUMS.FNM.REC.1400.117. In the first meeting, the participants signed the informed written consent after explaining the objectives of the research and the method of intervention and answering the questions of the participants.

### 2.5. Randomization

This study was based on a randomized design with two groups of intervention and placebo with a block randomization method with 4 blocks because this method minimizes sampling error and strengthens the power of statistical tests.

### 2.6. Sample Size Calculation

The sample size was calculated to compare changes in weight gain between the two groups based on Yeh et al.'s study [26]. Considering the confidence level of 95% and the test power of 80%, the sample size was calculated to be 59 people, which, considering a 20% drop out of the sample during the study, the sample size of seventy-one people in each group was calculated.

The sample size calculation formula is as follows:

(1) $$\begin{array}{c} n=\frac{{\left({Ζ}_{1-\left(\propto /2\right)}+{Ζ}_{1-\beta }\right)}^{2}\times \left(\delta_{1}^{2}+\delta_{2}^{2}\right)}{{\left(\mu_{1}-\mu_{2}\right)}^{2}},\\ n=\frac{{\left(1.96+0.84\right)}^{2}\times \left(2.06^{2}+2^{2}\right)\approx 59}{{\left(1.02\;–\;2.08\right)}^{2}}\text{.} \end{array}$$

### 2.7. Intervention

**Figure 1:**
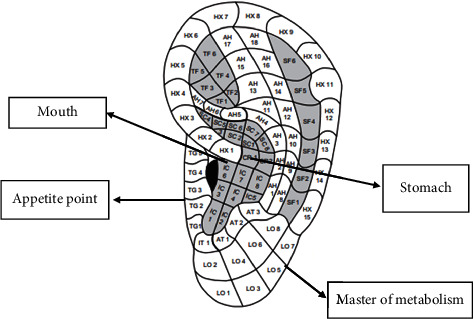
Location of seeds on both ears to control weight gain.

After providing explanations to familiarize the participants with the working method and answering their questions, the participants were requested to be comfortable. The first author who has an auriculotherapy certificate did the intervention. The intervention began by disinfecting both ears with a 70% alcohol solution. After determining the location of metabolism and stomach points in the left ear and mouth and appetite points in the right ear related to weight and appetite control, the researcher placed the seeds on the desired points.  Figure 1 shows the location of the seeds on both ears. The intervention lasted for a total of 5 weeks. The seeds were changed twice a week (once every three days) by the researcher. The participants in the intervention group were taught to press the seeds 6 times a day for one minute each time. The pressure method was to use moderate stimulation with continuous pressure. In the first session, the researcher fully taught the participants the amount of pressure and the duration of it in a practical way and asked them to do this once in her presence to ensure that it was correct. Participants were recommended to do this preferably 20 minutes before eating [27]. The researcher reminded the participants in the intervention group of their daily interventions by phone or text message. Each night, they were asked to check if they had followed the instructions and completed the daily registration checklist. In each seed replacement session, which was performed every three days, the checklist of the previous session was viewed and checked, and a checklist was received every week at the same time as the participants were weighed. Subjects were also emphasized in case of any symptoms of allergies or infections and pain as soon as possible through the contact number provided to them to discuss the issue with the researcher to remove the seeds.

In the placebo group, instead of real seeds, a label without *Vaccaria* seed (waterproof fabric adhesive) was placed by the researcher at the desired points in both ears, and the participants did not receive training to compress the points. They also did not receive the list of daily pressing points. All follow-ups and replacement of labels were performed in the same way as the intervention group in the placebo group. Finally, all participants were requested to notify the researcher if any seeds or labels were removed for any reason. It should be noted that pregnant mothers were unaware of the nature of the group to which they belonged.

### 2.8. Outcome Measures

At first, the demographic characteristics of the participants were completed by a researcher-made questionnaire including information such as age, education, job, number of pregnancies, weight before intervention, gestational age, parity, and BMI at the beginning of pregnancy. When the mothers visited, women in each center were weighed using a digital scale. This digital scale has a minimum capacity of zero and a maximum of 150 kilograms (kg) and has an accuracy of 0.1 kg. Height was measured with a portable stadiometer with a maximum permissible error of 0.5 cm between two measurements. All anthropometric measurements were performed by the researcher and were standardized according to the recommended instructions.

#### 2.8.1. Primary Outcomes

Our primary outcome was the average weight gain rate immediately after the end of the intervention between the intervention and control groups..

#### 2.8.2. Secondary Outcomes

Our secondary outcome was the average weight gain rate 2 and 4 weeks after the intervention between the two groups.

### 2.9. Safety of the Participants

Adverse events during treatment and follow-up periods were documented, reported to the ethics committee, and treated with appropriate treatment.

### 2.10. Data Analysis

Software SPSS version 22 was used to analyze the data. Descriptive statistics including mean, standard deviation, number, and percent were used to analyze the sample. Independent *T*-test, chi-square, and repeated measure ANOVA (analysis of variance) test were used to check the research objectives and test the hypotheses. The significance level was set at less than 0.05.

## 3. Results

**Figure 2:**
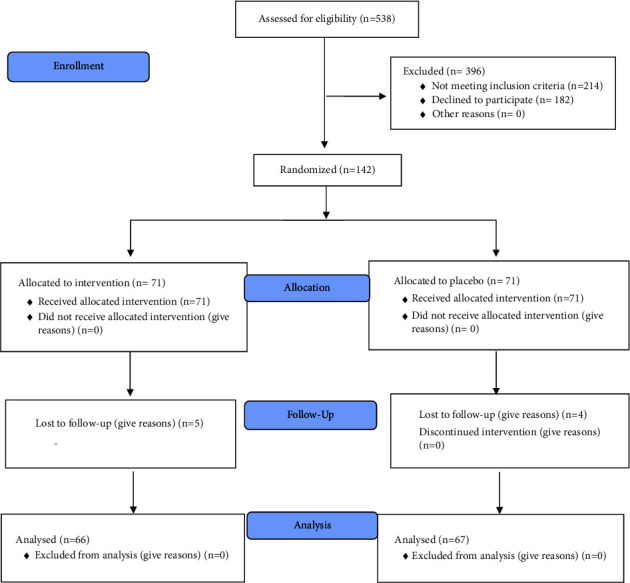
Flow diagram of the study.

The flowchart of the study is shown in Figure 2. A total of 538 pregnant women were examined for eligibility, of which 396 were excluded, and randomization was performed on 142 pregnant women (71 in each group). Sample dropout was five people in the intervention group and four in the placebo group due to lost follow-up visits. Finally, statistical analysis was performed on 66 people in the intervention group and 67 in the placebo group.

The average age of the participants was 25.0 ± 1.33. Most women were housewives and had a bachelor's degree or lower. During registration to the study, the mean gestational age of participants was 22.24 ± 1.28 weeks with an average BMI of 27.44 ± 1.42 kg/m^2^. There were no statistically significant differences between the intervention and placebo groups in demographic and obstetric characteristics (Table 1).

The independent *t*-test result showed no significant difference in the average weight gain of pregnant women before the intervention in the intervention and placebo groups (*P* > 0.05). While immediately after completing the study, there was a statistically significant difference between the two groups (*P* < 0.05). Also, there was a significant difference in the weight gain of women two weeks after the intervention in the auriculotherapy and placebo groups with a statistically significant difference (*P* < 0.001). This difference was also significant four weeks after the intervention (*P* < 0.001) (see Table 2).

The results of the repeated measure ANOVA test are shown in Table 3. To check the assumptions of this test (sphericity and equality of variances), Mauchly's sphericity test and Lune's test were performed, and the results of these tests indicated that the assumptions of sphericity (*P* = 0.09) and equality of variances (*P* < 0.05) were established, respectively. Finally, the *F*-test with Geyser's greenhouse index was used. The results of the repeated measure ANOVA test for the within-group effects showed that the interaction effect of time and group is significant for the variable of weight gain.

## 4. Discussion

The present study was conducted to investigate the effect of ear acupressure (auriculotherapy) on the weight gain of overweight women during pregnancy. This study showed that auriculotherapy controls natural weight gain during pregnancy. Weight in the intervention group immediately two and four weeks after the end of the intervention was significantly less than that in the placebo group, indicating that this method had more than just momentary effects.

To the best of our knowledge, no study assessed the effect of auriculotherapy on weight gain of pregnant women, whereas there are several papers on nonpregnant individuals. So, we discussed it with nonpregnant women. In a randomized controlled study, Kim et al. have conducted a study on the effect of auriculotherapy on obesity and self-efficacy of college girls. In this study, 49 girls were divided into two groups: experimental (25 people) and control (24 people). The intervention group received acupressure in five auricular points (Shenmen, mouth, stomach, endocrine, and small intestine points) for 4 weeks and was compared with the control group. Then, the body weight and BMI were checked between the two groups. Acupressure was effective in reducing weight and BMI of girls with obesity [28]. The results of the present study are in line with the results of that study, even though the research population was women but not pregnant and there was a difference in some pressure points of the ear. There is also a study by Fujimoto et al. in Japan. The results showed that auriculotherapy caused a significant reduction in people's weight. Also, auriculotherapy reduced the consumption of snacks and accelerated satiety [29]. The results of Fujimoto's research are consistent with the results of the present study, despite the nonpregnant research population and the different methods of doing the intervention.

A systematic review and meta-analysis by Mendonça et al. evaluated the effects of auriculotherapy on weight loss or BMI in nonpregnant patients with overweight or obesity. The most common stimulation points in the reviewed studies were the stomach, hunger, and mouth points, which are similar to our study. The duration of these studies ranged from 4 weeks to 3 months. Meta-analysis showed that BMI reduction in the auriculotherapy group was significantly higher than that in the placebo group [20]. The results of this study, despite the nonpregnant research population, in terms of ear stimulation points, the average duration of intervention, and the effectiveness of auriculotherapy on the weight variable, are in line with our study.

Lillingston et al. conducted a randomized controlled trial aiming at providing clarity on the effect of auriculotherapy on weight loss. The intervention was carried out for seven sessions a week, and waist circumference, weight, and mood changes were examined in both placebo and intervention groups. In this study, the therapeutic effect of auriculotherapy for weight loss and BMI was not significant [30], which was not consistent with our study. This negative result may be related to different points of stimulation and dissimilar methods of stimulation of points. Also, Cha and Park have conducted a study titled “The Effect of Auriculotherapy on Adolescent Obesity.” In this study, 58 teenagers with obesity were divided into two groups: experimental (32 people) and control (26 people). The intervention group received acupressure using *Vaccaria* seeds, and the control group received a placebo for eight weeks. Weight, BMI, and total cholesterol were assessed after intervention. Ear pressure could not decrease the body weight and BMI statistically. However, it could reduce the total cholesterol and low-density lipoprotein cholesterol levels in adolescents with obesity [31]. This inconsistency could be due to the small number of samples in that study, which could not be statistically significant despite the weight loss in the intervention group compared to the control group.

## 5. Limitations

Self-reporting by mothers was one of the limitations of this research. Since the women performed ear acupressure and filled the daily checklist sheets related to pressing the ears, the honesty and correctness of the research units were not in our control. However, the researcher asked the subjects to mark the corresponding box only if they pressed the keys every time, and during the study, she tried to gain trust by establishing proper and effective communication and making phone calls or text messages with the subjects while reminding them of the importance of the research. It caused this limitation to be minimized.

## 6. Conclusions

The results showed that auriculotherapy or ear acupressure was effective in controlling weight gain and caused a decrease in excessive weight gain in overweight pregnant women. The results of the present study indicated the effectiveness of auriculotherapy by stimulating the points of the mouth, appetite, stomach, and metabolism in controlling the weight gain of pregnant women. Because auriculotherapy did not have any side effects, this treatment could be a safe method for the mother, fetus, and baby with easy access and low cost for people with low-risk pregnancies.

## Figures and Tables

**Table 1:** Demographic and obstetrics characteristics of overweight pregnant women in the auriculotherapy and placebo group.

Characteristics		Placebo (*N* = 67)	Auriculotherapy (*N* = 66)	*P* value
		Mean ± SD	Mean ± SD	
		*N* (%)	*N* (%)	
Weight before intervention (kg)		79.63 ± 7.07	81.94 ± 7.47	0.540^*∗*^
Gestational age (weeks)		21.88 ± 1.43	22.60 ± 1.14	0.063^*∗*^
Parity (*n*)		1.03 ± 0.816	0.71 ± 0.718	0.590^*∗*^
BMI at the beginning of pregnancy (kg/m^2^)		27.15 ± 1.58	27.73 ± 1.27	0.13^*∗*^
Age (y)	18–25	22 (32.8)	15 (22.7)	0.16^*∗∗*^
	26–30	22 (32.8)	25 (37.9)	
	31–35	13 (19.4)	21 (31.8)	
	36–40	10 (14.9)	5 (7.6)	
Education	Under diploma	39 (58.2)	29 (43.9)	0.12^*∗∗*^
	Diploma to bachelor	28 (41.8)	35 (53.0)	
	Master's degree	0 (0.0)	2 (3.0)	
Job	House wife	54 (80.6)	57 (86.4)	0.523^*∗∗*^
	Employed	10 (14.9)	8 (12.1)	
	Other	3 (4.5)	1 (1.5)	
Number of pregnancies	1	23 (34.3)	29 (43.9)	0.280^*∗∗*^
	2	27 (40.3)	27 (40.9)	
	≥3	17 (25.4)	10 (15.2)	

^*∗*^Independent *t*-test; ^*∗∗*^chi-square test.

**Table 2:** Comparison of average weight gain of pregnant women in auricolotherapy and placebo groups in different periods.

Variable (weighing)	Group					
	Auricolotherapy		Placebo		Independent *t*-test statistic	*P* value
	Mean	SD	Mean	SD		
Before the intervention (g)	2944.82	338.33	2854.83	452.13	2.34	0.3
Immediately after the intervention (g)	1120.68	425.83	2704.09	344.96	−11.21	0.018
Two weeks after the end of the intervention (g)	793.10	278.38	1090.32	330.31	−16.42	<0.001
Four weeks after the end of the intervention (g)	729.31	241.52	964.51	348.35	−13.283	<0.001

**Table 3:** Pairwise comparison of the difference in weighting scores of women in two auricolotherapy and placebo groups by adjusting the effect of age, gestational age, body mass index, number of pregnancies, and delivery at different measurement times.

Source of variation	SS	df	MS	*F*	*P* value
Between groups	39803092.0	1	320382647.0	247.0	<0.001
Within groups	38447715.00	1	34182317.00	17.08	0.008
Error	275715951.1	133	4753723.000		
Total	186780185.01	133	3220348.024		

## Data Availability

The datasets used and/or analyzed during the current study are available from the corresponding author on reasonable request.

## Abbreviations

WHO: World Health Organization
BMI: Body mass index
IOM: Institute of Medicine
TUMS: Tehran University of Medical Sciences
ANOVA: Analysis of variance.
